# Restrictive filling patterns in patients with reduced systolic left ventricular function: identification by velocity encoded magnetic resonance imaging

**DOI:** 10.1186/1532-429X-13-S1-P176

**Published:** 2011-02-02

**Authors:** Kai Muellerleile, Loant Baholli, Michael Groth, Achim Barmeyer, Gerhard Adam, Gunnar K Lund, Thomas Rostock, Ulf K Radunski, Ralf Koester, Stephan Willems

**Affiliations:** 1 University Medical Center Hamburg-Eppendorf, Hamburg, Germany; 2 Klinikum Dortmund, Dortmund, Germany

## Purpose

To evaluate the ability of velocity encoded magnetic resonance imaging (VENC-MRI) to identify the presence of a restrictive filling pattern in patients with reduced systolic left ventricular (LV) function.

## Introduction

A restrictive filling pattern is an independent prognostic marker for an increased mortality in patients with reduced systolic LV function. The diagnosis is currently established by characterization of transmitral and pulmonary-venous flow using Doppler-echocardiography. VENC-MRI enables robust quantification of transmitral as well as pulmonary-venous flow.

## Methods

**Figure 1:**
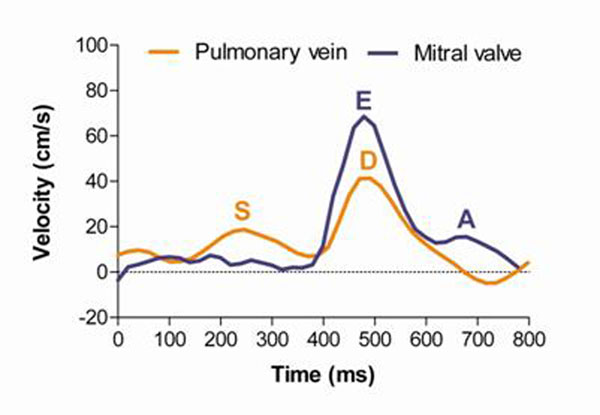
Transmitral and pulmonary venous flow characteristics by VENC-MRI

The study included 41 patients with reduced systolic LV function (ejection fraction 29±12 %). All patients underwent VENC-MRI and Doppler-echocardiography to assess the transmitral and pulmonary-venous flow characteristics. Figure 1 illustrates measurements of maximal early- and late-diastolic transmitral velocities (E- and A-waves) as well as maximal systolic and diastolic pulmonary venous velocities (S- and D-wave). Restrictive filling pattern was defined by an E/A ratio > 2.0 in combination with an S/D ratio < 1.0. Left atrial volume was obtained on long-axis cine-MRI slices using the biplane area-length method. N-terminal pro brain natriuretic peptide (NT-proBNP) levels were assessed as a marker for changed filling pressures. Maximal oxygen uptake (VO2-max) was assessed using spiroergometry.

## Results

There was a very good correlation between VENC-MRI and Doppler-echocardiography for the E/A ratio (r=0.86, P<0.0001). The correlation was moderate between both methods for the S/D ratio (r =0.45, P<0.01). VENC-MRI identified 10 (24 %) and Doppler-echocardiography 7 (17 %) patients with restrictive filling pattern. The agreement between both methods was moderate (kappa= 0.49). Left atrial volumes were larger in patients with restrictive filling pattern than in patients without restrictive filling pattern (143±41 vs. 104±33 ml; P<0.01). Higher NT-proBNP levels were found in patients with restrictive filling pattern compared to patients without restrictive filling pattern (6090±7854 vs. 1193±1387 ng/l; P<0.01). VO2max was lower in patients with restrictive filling pattern compared to patients without restrictive filling pattern (11.2±2.3 vs. 14.2±4.8 ml/min/kg; P=0.13)

## Conclusions

VENC-MRI has the ability to identify the presence of a restrictive filling pattern and may be a useful tool for the evaluation of patients with reduced systolic LV function.

